# Knowledge, Attitudes, and Clinical Experience with Cannabinoid Therapies: A Cross-Sectional Survey of Rheumatologists

**DOI:** 10.31138/mjr.310825.hct

**Published:** 2026-05-13

**Authors:** Ahmed Mougui, Zineb Baba, Imane El Bouchti

**Affiliations:** Department of Rheumatology, Arrazi Hospital, Mohammed VI University Hospital, Faculty of Medicine and Pharmacy, Cadi Ayyad University, Marrakesh, Morocco

**Keywords:** cannabinoids, surveys and questionnaires, rheumatologists, knowledge, confidence

## Abstract

**Aim:**

In Morocco, cannabinoid therapies have recently been legalised. This study aimed to explore the knowledge, attitudes, and perceptions of Moroccan rheumatologists toward these emerging treatments.

**Methods:**

A pretested online questionnaire was distributed via email to all members of the Moroccan Society of Rheumatology. The survey assessed participants’ knowledge, prescribing confidence, perceived risks, safety precautions, and barriers to the use of cannabinoid therapies.

**Results:**

Of the 400 rheumatologists contacted, 126 responded (response rate: 32%). Among them, 59.5% reported confidence in their knowledge of cannabinoid therapies. A total of 80.6% of respondents believed that pharmacological cannabinoids may have a role in managing rheumatic diseases, while 9.7% declared that there is currently no therapeutic role for cannabinoids in rheumatology. Only 7.1% had recommended a therapeutic trial, and 52.8% expressed concern that the use of cannabinoids may cause patients to discontinue effective standard treatments. Fibromyalgia was the most frequently cited indication (85.7%). The main barriers to prescribing cannabinoid therapies were concerns about a history or risk of substance abuse (79.2%) and patient non-compliance with medical advice (79.2%).

**Conclusion:**

This finding highlights hesitation and caution toward these emerging treatments in Morocco. There is a clear need for evidence-based guidance to support rheumatologists in clinical decision-making concerning cannabinoids.

## INTRODUCTION

Pain associated with rheumatic diseases is often chronic and frequently accompanied by fatigue, as well as mood and sleep disturbances. Current drugs to treat pain offer only modest effects, and are commonly associated with side effects.^1^ In response to these limitations, patients are seeking alternative solutions to manage their pain. Cannabinoid therapies have recently emerged as a possible option and are gaining popularity among patients with rheumatic diseases.^2^ Rheumatologists, as the primary specialists managing these patients, must be sufficiently informed to advise their patients. However, surveys of rheumatologists in Western countries have revealed that rheumatologists lack confidence in their knowledge and skills related to prescribing cannabinoid therapies.^3,4^

Cannabinoid therapies were recently legalised in Morocco.^5^ This is likely to increase patient interest, prompting questions and expectations directed toward rheumatologists. To date, no study has investigated the perspectives of Moroccan rheumatologists on this topic. This study aims to explore the knowledge, attitudes, and perceptions of Moroccan rheumatologists regarding cannabinoid therapies.

## METHODS

We conducted a national cross-sectional online survey targeting all Moroccan rheumatologists working in both the public and private sectors. The email list was provided by the Moroccan Society of Rheumatology (SMR). This study was conducted in accordance with current recommendations for the design and reporting of survey studies.^6^

### Bibliographic Research and Questionnaire Development

The questionnaire was developed by a panel of four rheumatologists: two academic instructors and two practicing specialists, and was based on a literature review. This review identified two studies with similar objectives, conducted in different cultural settings ^3, 4^, which served as foundational references for the questionnaire’s design.

### Questionnaire Validation and Distribution

The initial version of the questionnaire was pretested with four rheumatologists. Their feedback focused on clarity, comprehensiveness, ease of completion, and the time required for completion. Based on their feedback, we revised the questionnaire.

The final version of the questionnaire comprised 18 items, structured into the following thematic sections (**Appendix 1**):

1. Socio-demographic and professional background
2. Knowledge, attitudes, and perceptions of cannabinoid therapies
3. Perceived risks, precautions, and barriers to prescribing cannabinoid therapies

The survey was implemented using Google Forms. It was distributed by email to all members of the SMR. Responses were collected anonymously over a four-week period, from 30 January to 28 February 2025.

### Ethics approval and informed consent

A brief introduction was provided at the beginning of the questionnaire, including detailed information about the study’s objectives, methodology, and assurances of anonymity and confidentiality. Completion and submission of the questionnaire were considered as valid informed consent.

This study was approved by the Ethics Committee of the Faculty of Medicine and Pharmacy of Marrakesh (Approval Number: 005/2025, approved on January 23, 2025).

### Statistical Analysis

Statistical analyses were performed using Jamovi version 2.6.24. Categorical variables were summarised using frequencies and percentages. Associations between categorical variables were assessed using the Chi-square test. No corrections for multiple comparisons were applied, as the analyses were exploratory in nature.^7^ A p-value < 0.05 was considered statistically significant.

## RESULTS

### Physician Demographics

The survey was circulated among the 400 members of the SMR, with 126 physicians responding, corresponding to a response rate of 32%.

Most participants were aged between 30 and 50 years, and 73.8% were women. A majority had more than 20 years of professional experience, and 42.9% were working in the private sector. Demographic characteristics of the participants are shown in **Table 1**.

**Table 1.** Demographic characteristics of the participants (n = 126).

**Characteristics**	**Number (%)**
**Age**	
30 or younger	17 (13.5)
30–50	67 (53.2)
50 and over	42 (33.3)
**Gender**	
Female	93 (73.8)
Male	33 (26.2)
**Practice Type**	
Public	21 (16.7)
Private	54 (42.9)
Academic	51 (40.5)
**Years in practice**	
<5	34 (27)
5–10	34 (27)
11–20	15 (11.9)
>20	43 (34.1)

### Physician Knowledge, Attitudes, and Perceptions of Cannabinoid Therapies

Regarding their current knowledge of cannabinoid therapies, 40.5% of participants reported a lack of confidence, 44.4% felt somewhat confident, and 13.5% felt confident.

Among the respondents, 80.6% reported that only pharmacologic cannabinoids have a therapeutic role in the management of rheumatic diseases, 0.8% believed that herbal cannabis alone has a role, and 8.9% considered that both pharmacologic cannabinoids and herbal cannabis could be used therapeutically. In contrast, 9.7% of participants believed that there is currently no therapeutic role for any form of cannabinoids in rheumatology.

Fibromyalgia was the most frequently cited indication for cannabinoid therapies in rheumatic diseases (85.7%), followed by low back pain (50.8%), osteoarthritis (46.0%), spondyloarthritis and rheumatoid arthritis (28.6% each), and gout (4.8%).

Most participants reported that they would consider prescribing cannabinoid therapies when conventional treatments fail (64.3%), while only 9.5% indicated they would do so solely at the patient’s request.

Among the 125 respondents, 52.8% believed that the use of cannabinoid therapies could lead patients to discontinue effective standard treatments. Only 7.1% had previously conducted a therapeutic trial of cannabinoid therapies, but 66.7% indicated that they would recommend one if indicated.

Nearly half of the respondents (48.4%) reported no confidence in prescribing cannabinoid therapies, including determining dosage, frequency, and route of administration, while 40.5% were somewhat confident and 10.3% were confident (**Table 2**).

**Table 2.** Physician knowledge, attitudes, and perceptions of cannabinoid therapies.

	**Number (%)**
**Confidence in Knowledge of cannabinoid therapies**	
Very confident	2 (1.6)
Confident	17 (13.5)
Somewhat confident	56 (44.4)
Not confident	51 (40.5)
**Confidence in prescribing cannabinoids**	
Very confident	1 (0.8)
Confident	13 (10.3)
Somewhat confident	51 (40.5)
Not confident	61 (48.4)
**Perceived role of cannabinoids in rheumatic diseases (n= 124)**	
Pharmacologic cannabinoids only	100 (80.6)
Herbal cannabis only	1 (0.8)
Both	11 (8.9)
No role	12 (9.7)
**Perceived indications for cannabinoid therapies**	
Low back pain	64 (50.8)
Fibromyalgia	108 (85.7)
Osteoarthritis	58 (46.0)
Spondyloarthritis	36 (28.6)
Rheumatoid arthritis	36 (28.6)
Gout	6 (4.8)
Others	14 (11.1)
**Conditions for considering the use of cannabinoid therapies**	
When conventional treatments fail (yes/no)	81 (64 .3) / 45 (35 .7)
Only at the patient's request (yes/no)	12 (9.5) / 114 (90.5)
Could the use of cannabinoids lead patients to discontinue effective standard treatments? (yes/no) (n= 125)	66 (52.8) / 59 (47.2)
Have you ever conducted a therapeutic trial of cannabinoids?	9 (7.1) / 117 (92.9%)
Would you recommend a therapeutic trial of cannabinoids?	84 (66.7) / 42 (33.3%)

### Perceived Risks, Precautions, and Barriers to Prescribing Cannabinoid Therapies

When asked about their main concerns regarding the use of cannabinoid therapies, the most frequently identified issues were: potential for abuse or misuse (91.2%), side effects (64%), lack of scientific evidence (54.4%), and drug interactions (43.2%).

Respondents reported the following precautions they would communicate to patients when prescribing cannabinoid therapies: the risk of excessive use or dependence (88%), the potential impact on work performance or safety (80%), possibility of adverse effects when combined with other medications (72.8%), the need to apply precautions similar to those used for opioid-based treatments (53.6%), and the risk of cognitive impairment as well as pulmonary effects associated with smoking cannabis (42.4%).

Regarding the main barriers to prescribing cannabinoid therapies, respondents most frequently cited the history or risk of substance abuse (79.2%), patient non-compliance with medical instructions (79.2%), the presence of mental health disorders (73.6%), and the risk of impaired alertness or vigilance (67.2%) (**Table 3**).

**Table 3.** Perceived risks, precautions, and barriers to prescribing cannabinoid therapies.

**Survey Question**	**Response, n (%)**
**What are your main concerns regarding the use of cannabinoids**?	
Side effects	80 (64)
Lack of scientific evidence	68 (54.4)
Abuse or misuse	114 (91.2)
Drug interactions	54 (43.2)
**Which precautions would you advise patients about when prescribing cannabinoids?**	
Potential impact on work performance or safety	100 (80)
Excessive use or dependence	110 (88)
Possibility of adverse effects when combined with other medications	91 (72.8)
Risk of cognitive impairment or pulmonary effects from smoking	53 (42.4)
Precautions similar to those used for opioid-based treatments	67 (53.6)
**What do you consider the main barriers to prescribing cannabinoids?**	
History or risk of substance abuse	99 (79.2)
Presence of mental health disorders	92 (73.6)
Patient non-compliance with medical instructions	99 (79.2)
Risk of impaired alertness or vigilance	84 (67.2)

### Grouping of respondents

#### According to Confidence in Knowledge of Cannabinoid Molecules

Among respondents who reported confidence in their knowledge of cannabinoid therapies, 86.7% believed that only pharmacological cannabinoids have a therapeutic role in rheumatic diseases, compared to 71.4% among non-confident respondents. In contrast, only 4% of confident respondents stated that cannabinoid therapies have no therapeutic role, versus 18.4% in the non-confident group. Furthermore, 81.3% of confident respondents reported confidence in their knowledge regarding the prescription of cannabinoid therapies, including dosage, frequency, and route of administration, compared to only 7.8% of those without confidence. When conventional treatments fail, 73.3% of confident respondents would consider using cannabinoid therapies, compared to 51% of non-confident respondents. Finally, 78.8% of confident respondents stated they would recommend a therapeutic trial of cannabinoid therapies if indicated, compared to 49% among non-confident respondents (**Table 4**).

**Table 4.** Grouping of respondents according to confidence in knowledge of cannabinoid therapies.

	**Confident (n= 75)**	**Not confident (n= 51)**	**p**
**Perceived Role of cannabinoid therapies**			
Pharma only	65 (86.7%)	35 (71 .4%)	0.04
Herbal cannabis only	0	1 (2%)	0.21
Both	7 (9.3%)	4 (8.2)	0.82
No role	3 (4%)	9 (18.4)	0.01
**Confidence in prescribing cannabinoids**	61 (81.3%)	4 (7.8)	<0.01
**Conditions for considering the use of cannabinoids**			
When conventional treatments fail	55 (73.3)	26 (51)	0.01
Only at the patient's request	10 (13.3)	2 (3.9)	0.08
**Have you ever conducted a therapeutic trial with cannabinoids?**	7 (9. 3%)	2 (3.9)	0.25
**Would you recommend a therapeutic trial with cannabinoids?**	59 (78.7)	25 (49%)	<0.01
**Could the use of cannabinoids lead patients to abandon effective standard treatments?**	40 (53.3)	26 (52)	0 .88

#### According to the Conduct of a Therapeutic trial with Cannabinoid Therapies

When respondents were divided into those who had conducted a therapeutic trial with cannabinoid therapies (n= 9) and those who had not (n= 117), no statistically significant differences were observed between the two groups in terms of:

-Their perception of the therapeutic role of cannabinoid therapies;
-Their confidence in their knowledge of cannabinoid therapies;
-Their confidence in prescribing cannabinoid therapies;
-The clinical situations in which they would consider its use;
-Their perception of the risk that patients might abandon effective conventional treatments (**Table 5**).

**Table 5.** Distribution of rheumatologists according to the conduct of a therapeutic trial with cannabinoid therapies.

	**Yes (n= 9)**	**No (n= 117)**	**p**
**Perceived Role of cannabinoid therapies**			
Pharma only	8 (88.9)	92 (80)	0.516
Herbal cannabis only	0	1 (0.9)	0.779
Both	1 (11.1)	10 (8.7)	0.806
No role	0	12 (10.4)	0.308
**Confidence in prescribing cannabinoid therapies**	7 (77.8)	58 (49.6)	0.103
**Conditions for considering the use of cannabinoids**			
When conventional treatments fail	8 (88.9)	73 (62.4)	0.110
Only at the patient's request	0	12 (10.3)	0.312
**Confident in knowledge**	7 (77.8)	68 (58.1)	0.247
**Would you recommend a therapeutic trial with cannabinoids?**	6 (66.7)	78 (66.7)	1
**Could the use of cannabinoids lead patients to abandon effective standard treatments?**	3 (33.3)	63 (54.3)	0.225

## DISCUSSION

This is the first survey assessing the knowledge and attitudes of Moroccan rheumatologists toward cannabinoid therapies. The results provide valuable insights into their perceptions and concerns, helping to provide a better understanding of the potential and challenges involved in integrating these emerging therapies into clinical rheumatology practice.

The results indicate that 59.5% of Moroccan rheumatologists are confident in their knowledge of these therapies. This percentage is significantly higher than those reported in Canada (25.8%) and Israel (26.1%).^3,4^ While surprising, this difference may reflect self-perceived competence rather than actual academic expertise. In contexts where cannabinoid research and clinical application are more advanced, practitioners may demonstrate greater caution and critical self-assessment regarding their knowledge.

In this study, 80.6% of respondents reported that pharmacological cannabinoids alone play a role in managing rheumatic diseases. These findings differ from those of Canadian and Israeli surveys. In Canada, 45% of rheumatologists reported that cannabinoid therapies have no therapeutic value, while in Israel, 74% indicated that both pharmaceutical cannabinoids and herbal cannabis could be beneficial.^3,4^ These discrepancies may reflect differences in sociopolitical, cultural, religious, clinical practice, and regulatory environments that shape physicians’ perceptions and attitudes.^8 –11^

If conventional analgesics fail, 64.3% of Moroccan rheumatologists reported that they would prescribe cannabinoid therapy, compared with 83% of Israeli rheumatologists and 28% of Canadian rheumatologists who appear more reluctant,^3,4^ possibly due to the lack of strong evidence in rheumatic diseases.^12^ Additionally, 9.5% of Moroccan rheumatologists indicated they would prescribe cannabinoid therapy at the patient’s request, regardless of prior treatments, a proportion similar to Israeli rheumatologists (9%) but higher than Canadian rheumatologists (4%). This finding is concerning, as current evidence does not support prescribing cannabinoid therapy based solely on patient demand without considering prior treatments. Conventional analgesics should be attempted first,^13,14^ and any prescription of cannabinoids requires careful evaluation of contraindications and potential drug-drug interactions.^15,16^

Fibromyalgia (85.7%), chronic low back pain (50.8%), and osteoarthritis (46.0%) were identified by respondents as the main conditions potentially warranting a prescription for cannabinoid therapy. These disorders are complex, characterised by chronic pain that is often insufficiently controlled by conventional treatments, the long-term use of which may also be associated with significant adverse effects. Furthermore, they are frequently accompanied by additional symptoms such as anxiety, fatigue, and sleep disturbances, which further strengthen the rationale for considering cannabinoids as an alternative therapeutic option.^17–19^

Only 7.1% of respondents had ever recommended a therapeutic trial of cannabinoids, compared to 66.7% of Israeli rheumatologists and 30% of Canadian rheumatologists.^3,4^ This discrepancy may be explained by the recent legalisation of cannabinoid therapies in Morocco, whereas in Israel and Canada, its therapeutic use has a longer tradition and benefits from more established clinical experience. Nonetheless, an evolution appears likely, as 66.7% of Moroccan respondents indicated that they would be willing to recommend a therapeutic trial in the future, a proportion comparable to that of Israeli rheumatologists (65%) and higher than that of their Canadian counterparts (40%).^3,4^ This readiness reflects the expectation among rheumatologists that cannabinoid therapies could help address some of the unmet therapeutic needs in the management of rheumatic conditions.

In our survey, 52.8% of respondents reported that cannabinoid therapies use may lead patients to discontinue effective standard treatments. This finding is consistent with a survey conducted in the United States and Canada, where nearly two-thirds of individuals with rheumatic diseases reported substituting commonly prescribed medications for symptom control with cannabinoid therapies. Alarmingly, 37 patients reported replacing disease-modifying antirheumatic drugs with cannabinoid therapies, raising serious concerns about potential disease flare-ups and loss of long-term disease control. Furthermore, the survey revealed additional worrisome patterns: inhalation was the most common mode of administration, THC-based products were the most widely used, and patients who replaced conventional treatments with cannabinoid therapies tended to use it more regularly and over longer periods.^20^

The precautions, hesitations, and concerns expressed by Moroccan rheumatologists regarding cannabinoid therapies are consistent with those reported by their Canadian and Israeli counterparts,^3,4^ reflecting an international convergence of rheumatologists’ reservations toward the use of cannabinoid therapies.

Among Moroccan rheumatologists who reported confidence in their knowledge of cannabinoid therapies, 86.7% believed that cannabinoids have a therapeutic role in rheumatic diseases, compared to 30% of Canadian rheumatologists. Conversely, only 4% of confident Moroccan respondents considered cannabinoid therapies to have no therapeutic role, compared to 21% of Canadians. In terms of clinical practice, 81.3% of confident respondents felt capable of prescribing cannabinoid therapies, whereas this was reported by 27% of Canadian rheumatologists. Furthermore, 73.3% indicated they would consider cannabinoid therapies when conventional treatments fail, compared to 42% in Canada. Finally, 78.8% of confident respondents reported that they would recommend a therapeutic trial of cannabinoid therapies when indicated, compared to 66% of Canadian rheumatologists.^4^

This study has several limitations. First, its cross-sectional design. Second, the relatively low response rate (32%) may introduce a risk of non-response bias. However, this rate is comparable to those reported in similar surveys among rheumatologists (25% in Canada and 19.3% in Israel). Several factors may explain the non-response, including limited familiarity or comfort with the topic of cannabinoid therapies, disagreement with their use in rheumatic conditions, cultural or religious sensitivities related to medical cannabis, or general apathy toward survey participation. Additionally, it is difficult to determine whether the respondents are fully representative of the broader population of Moroccan rheumatologists, as detailed national demographic data were not available. Third, participation was voluntary, which may have introduced selection bias, favouring responses from individuals with stronger opinions or a particular interest in the subject. Fourth, all findings are based on self-reported knowledge, perceptions, and attitudes, which may not necessarily reflect actual clinical expertise or practice behaviours. Additionally, comparisons with similar studies conducted in Canada and Israel should be approached with care, given the significant differences in cultural norms, legal frameworks, and regulatory environments surrounding medical cannabis. Such factors likely influence clinicians’ views and may limit the generalisability of cross-country comparisons.

## CONCLUSIONS

The results of this study indicate that approximately 60% of Moroccan rheumatologists express confidence in their knowledge of cannabinoid therapies. However, only a very small number have ever conducted a therapeutic trial involving these treatments. This reflects a degree of hesitation and caution regarding these emerging therapies in Morocco, despite significant media coverage that has contributed to growing interest among patients with rheumatic diseases. This situation increases the pressure on rheumatologists, who are expected to stay up to date with the current state of scientific knowledge on cannabinoid therapies.

## Data Availability

The datasets are available from the corresponding author on reasonable request.

## APPENDIX 1

https://mjrheum.org/assets/files/MJR%202025-0299_Appendix%201.pdf
